# Punitive Disentitlement Within Private Law?

**DOI:** 10.1093/ojls/gqaf004

**Published:** 2025-03-05

**Authors:** Timothy Liau

**Keywords:** punishment, private law, disentitlement, remedies, culpability

## Abstract

Does private law punish? Should it? I question whether private law punishes in a form other than through a court order of punitive damages, by exploring a less obvious form of punishment to which less attention has been paid—‘punitive disentitlement’—wherein a person is disentitled from a legal right, defence, or other legal advantage they would and should otherwise be entitled to, because of their misconduct. Potential instances are identified and analysed in a broad survey of private law doctrine, including the laws of property, contract, unjust enrichment and torts. The strongest reason for punitive disentitlement is its immunity to a powerful normative objection to punitive damages. Punitive disentitlement is not free from difficulties, however. It inherits some of the difficulties associated with punitive damages; it also runs into a separate set of objections. We should therefore be more alert to, and cautious about, its continued use.

## 1. Introduction

Does private law—understood conventionally as including the laws of contract, torts, unjust enrichments and property—punish its subjects for their alleged misconduct by disentitling them from legal rights, defences, or other legal advantages they would and should otherwise be entitled to under its norms? I believe there is a plausible case for answering ‘yes’, and I call this ‘punitive disentitlement’. My argument is presented in three parts.

The first part consists of conceptual ground-clearing. It is comprised of sections 2 and 3. The idea of disentitlement, and how it could potentially constitute a form of punishment, is introduced. It should be noted upfront that what I call ‘disentitlement’ has been referred to interchangeably as ‘disqualification’, and sometimes ‘forfeiture’. My preferred terminology is ‘disentitlement’ because it better captures the broader idea that disentitlement can occur anticipatorily, through preclusion. Through the operation of private law rules and norms, a person could be excluded from obtaining something to which they should and would otherwise be entitled, but which they may not yet have acquired. The language of ‘forfeiture’ or ‘disqualification’ might, by contrast, be mistaken as confined only to the deprivation of something they already have, a narrower idea.

The second part, section 4, examines select case law examples from across the doctrinal terrain. The aim is not to enumerate exhaustively all possible cases. It is to raise a number sufficient to invite greater reflection on the possibility that private law might punish, albeit in a manner or sense different than conventionally assumed.

The third part, section 5, addresses normative implications and the justifiability of punitive disentitlement as a practice. It asks whether punitive disentitlement is open to the same normative objections as punitive damages. It also asks whether there are objections uniquely associated with punitive disentitlement, considering what this means for its future.

My overall claim is that disentitlements *can* be punitively motivated, and that there is evidence of this within private law doctrine. Their chief advantage is in being immune to the most forceful, and to my mind unanswerable, normative objection to punitive damages: the illegitimate conferral of legal entitlements to private individuals. We should not be so quick to endorse its expansion, however. Punitive disentitlement faces other objections which caution against its widespread use. It is necessary in each case to ask whether disentitling someone as a means of punishing them is compatible with the moral constraints on legitimate punishment more generally, and this requires clearer and better articulation of the relevant ‘misconduct’ that private law norms are already implicitly responding to.

This article thus seeks to contribute to the debate on the role and reach of punishment within private law by (i) exploring a phenomenon to which little attention has been paid; (ii) showing that it may be more prevalent than assumed; (iii) prompting much-needed reflection on use of the terms ‘punitive’ and ‘punishment’ in judicial legal reasoning; and (iv) opening up further inquiry about other overlooked instances.

## 2. Disentitlement as a Form of Punishment

There may be more species of punishment than one. An unexamined assumption that punitive damages is the sole mode of punishing within private law may have blinkered our view to the wider landscape.

In the literature debating the legitimate role and scope of punitive measures within private law, it appears almost instinctive to latch onto punitive damages as our paradigm example. The bulk of academic energy has been poured into debating one big question: can a private law damages award legitimately be used as a means of punishing a defendant for his civil wrong (ie a tort, breach of contract, or equitable wrong)?^1^

Views have ranged from abolitionism to expansionism. At the anti-punitive end of the spectrum it has been argued that, if punitive damages are motivated only by a desire to deter future tortfeasors or contract-breakers through making an example of the defendant, then ‘punishment is foreign to the structure of private law’, because doing so would be inconsistent with fundamental features of private law.^2^ On the pro-punitive end, advocates have argued that despite ‘illogicalities in the present law’, punitive damages ought to be retained, and their ambit extended.^3^

It seems questionable, however, whether liability to a court order of punitive damages is the *only* form of punishment that could occur within private law doctrine. The late John Gardner once argued that:

Friends, colleagues, spouses, siblings, and business partners regularly punish each other for actual or supposed wrongs that are not legal wrongs. They typically do so by withdrawing favours or cooperation, but there are many other possible ways, some of which are capable of involving the infliction of grave suffering. It is very common for one estranged spouse to punish the other, for example, by preventing him or her from spending time with his or her children, fully intending that this should be a terrible experience. I know of no reason to think that such punishment is ‘sub-standard or secondary’ as compared with, say, imprisonment by the courts.^4^

Could disqualifications be punitive? Although private lawyers have to my knowledge never given much consideration to this question, the notion is not entirely novel to the company or criminal lawyer. In *R v Steven Kenneth Young*, the Court of Appeal said that the offender’s disqualification from acting as a company director for two years under the Company Directors Disqualification Act 1986 was ‘unquestionably a punishment’.^5^ In his book *Why Punish?*, Nigel Walker comments that ‘a shared conception of punishment’ involves the infliction of something assumed to be unwelcome to the recipient, such as ‘the inconvenience of a disqualification, the hardship of incarceration’, etc.^6^

Consider also disentitlements following a criminal conviction. In the past, this could entail so extensive a loss of civil rights as to constitute ‘civil death’, a ‘form of punishment … [which] extinguished most civil rights of a person convicted of a crime and largely put that person outside the law’s protection’.^7^ Convicted persons would be deprived of the right to hold or transfer property, to vote, to bring suit in the courts, or even to make public statements or to visit certain places.^8^ Today, state-imposed disentitlements still exist in the form of disenfranchisement,^9^ disqualifications from driving,^10^ from entering public houses,^11^ from attending football matches,^12^ from keeping animals,^13^ etc. As a form of ‘invisible punishment’, their continued use has sparked debate.^14^

## 3. Conceptual Preliminaries

Does punishment through disentitlement occur within private law? A few clarifications are in order.

### A. Disentitlement versus Non-entitlement

My focus is on disentitlement rather than non-entitlement. The line may not always be easy to draw, but the conceptual difference is a real one, and can be illustrated here by examples.

A Ruritanian citizen is not entitled to vote in the 2025 Singapore General Elections. This is because she does not positively satisfy the eligibility conditions for the right to vote. Only Singaporean citizens of age who are ordinarily resident in Singapore are entitled to vote;^15^ she is not a Singaporean citizen; ergo she is not entitled to vote.

Contrast a Singaporean citizen who, even though of age and ordinarily resident in Singapore (ie satisfying the eligibility conditions), has been struck off the register of voters. Perhaps he has been struck off for failing to vote in a previous election, for taking an oath of allegiance to a foreign power or state, for having been convicted of a corrupt or illegal practice, or because he is currently a prisoner serving a custodial sentence exceeding 12 months.^16^

Due to the presence of these extra facts, this person has been disentitled from voting. That is an example of disentitlement rather than mere non-entitlement.

### B. Punitive versus Non-punitive Disentitlement

Not all instances of disentitlement are necessarily punitive.^17^ We are interested in whether *some* instances within private law doctrine, are. Like all punishments, punitive disentitlements are *responses*. They are triggered by the presence of extra facts: some form of ‘bad behaviour’ or ‘misconduct’ on the part of the person being disentitled.

To illustrate, suppose all Russians are disqualified by the International Olympic Committee from participating in the next Olympic games. Is this punishment, ie is such disentitlement, ‘punitive’? Possibly. Especially if we are then told that it is meted out in response to a past finding of systematic doping to gain an unfair competitive advantage, or, perhaps, in reaction to the invasion of a neighbouring country. Other potential examples include being refused entry into public places, onto public transport, dining out in a restaurant, or even a spot to compete at the Australian Open because one refuses to undergo state-mandated vaccination during a global pandemic.^18^

### C. For What Disentitled versus From What Disentitled

It is necessary, therefore, to keep separate two conceptually distinct aspects of our inquiry, even though they are related because one occurs in response to the other.

1. The first aspect—‘*for what* is one disentitled?’—identifies more specifically the relevant ‘misconduct’ to which private law rules and doctrines are responding, locating the trigger or ground for disentitlement. (As we shall later see in section 4, that is sometimes not easy to articulate precisely.)
2. The second aspect—‘*from what* is one disentitled?’—is an inquiry as to the legal advantage an offender is being deprived of. Is one being disentitled from a legal right? Is one being disentitled from a defence? Or is one being disentitled from the protection of some applicable legal rule or doctrine? Different modes of disentitlement could exist.

The first aspect is a question about *what* private law punishes; the second, a question about *how* private law punishes.

### D. Fault and Culpability

It is sometimes said, especially in the criminal law, that one ought not punish in the absence of fault. *Nullen poena sine culpa.* It bears reminding that this is a moral constraint, rather than an analytical truth.^19^

Punishment of the faultless can (and sadly does) occur. That does not however mean that it ceases to be punishment. Undeserved punishment still is punishment. Indeed, it is precisely because we recognise punishment of the innocent *as* punishment that we quarrel about whether such punishment is deserved or not.

It follows that disentitlement in the sense I am interested in does not necessarily entail that the person disentitled is at fault. This is a moral basis for criticising its appropriateness, discussed in section 5.

### E. ‘Punitive’

Defining ‘punishment’ is no easy task. Numerous philosophers have made countless attempts.^20^ Yet, disagreement persists. Partly, this is linguistic and conceptual; the term is vague and open-textured.^21^ Partly, it is irreducibly moral.

As a relatively neutral starting point, I adopt HLA Hart’s definition in this article, subject to a small revision.^22^ I shall count as ‘punishment’:

1. The infliction of hard treatment.
2. By an authority (or its representatives).
3. In response to alleged ‘misconduct’ by an ‘offender’.
4. Intended at^23^ deterrence, retribution or censure (or some mixture of aims thereof).

Lacey observes that ‘It is all too easy to allow definition to serve a covert normative function’. An overly narrow definition builds in assumptions which ought to be argued for openly.^24^ To avoid that, I added criterion 4. It is meant broadly to track the three main contrasting approaches towards justifying punishment: by aims of forward-looking consequentialism (‘deterrence’), backward-looking retributivism (‘retribution’) or expressivism (‘censure’),^25^ leaving room for hybrids.

There is, as Barker helpfully shows, a locus of disagreement around criterion 4, contributing to definitional and doctrinal instability.^26^ For example, landmark punitive damages cases like *Whiten v Pilot Insurance* say ‘their purpose is … to give a defendant his or her just desert (retribution), to deter the defendant and others from similar misconduct in the future (deterrence), and to mark the community’s collective condemnation (denunciation) of what has happened’.^27^ Yet compare another landmark case, *Cavendish v Makdessi*, declaring that ‘the rule against penalties is a rule of contract law based on public policy … that the courts will not enforce a stipulation for punishment for breach of contract’,^28^ the Supreme Court obviously assuming some aim other than deterrence.^29^

If one believes private law is foundationally relational, then judicial reasoning relying on ‘public policy’ and making examples of litigants as mere means to public-facing ends like general ‘deterrence’ will evoke punitive ideas, sitting uneasily within private law.^30^ For it does not prioritise the parties’ relations over broad societal arrangements, or the public.

But illegitimate punishment still *is* punishment. Punishment could be illegitimate, because ‘justified’ by unsatisfactory ends. That does not mean it ceases to be punitive thereby. That is why we can—indeed, must—interrogate its legitimacy. To assert otherwise, HLA Hart astutely observed, is an abuse of definition.^31^

## 4. Private Law Doctrine

Does ‘punitive disentitlement’ exist within private law? This section identifies and analyses potential instances. To support generalisation a wide enough range must be covered. Various examples from across private law doctrine are broadly surveyed. Given space constraints my coverage is necessarily illustrative, rather than exhaustive.

It will be seen that the labels ‘disentitlement’, ‘disqualification’, and ‘forfeiture’ have been used interchangeably. The reasoning is often unabashedly punitive.

### A. Property Rights and ‘Unlawful Killing’

Consider first an example from within property law, understood here to include succession and trusts. Before 1870 the property of a convicted felon was forfeited to the Crown.^32^ A modern instance of punitive disentitlement can be found in what is now called the ‘forfeiture rule’, a common law rule developed after forfeiture to the Crown was abolished.^33^

*Cleaver v Mutual Reserve Fund Life Association* is the seminal case.^34^ A beneficiary under a life insurance policy murdered her husband—the insured—by poisoning him. It was unanimously held that any statutory trust in the wife’s favour^35^ had ‘either never arisen or it has, by the act of the cestui que trust, become incapable of enforcement’.^36^ Thus, the wife could not claim payment of the insurance money.

Fry LJ articulated a ‘principle of public policy’ which ‘disqualifies [the wife] from asserting that she is the cestui que trust…’.^37^ He held that

no system of jurisprudence can with reason include amongst the rights which it enforces rights directly resulting to the person asserting them from the crime of that person. If no action can arise from fraud, it seems impossible to suppose that it can arise from felony or misdemeanour.^38^

This ‘principle of public policy’ quickly became well established.^39^*Re Crippen* expressed it as one that ‘no person can obtain, or enforce, any rights resulting to him from his own crime’;^40^*Re Hall*, as ‘a man shall not slay his benefactor and thereby take his bounty’.^41^ By *Re Giles*, it was said that ‘the cases have established beyond question that a person so convicted of manslaughter is disqualified from taking a benefit under the will or intestacy of the person whom he has killed’.^42^

This principle has since been statutorily restated in the Forfeiture Act 1982, which defines the ‘forfeiture rule’ as ‘the rule of public policy which in certain circumstances precludes a person who has unlawfully killed another from acquiring a benefit in consequence of the killing’.^43^

#### (i) From what disentitled

The ‘forfeiture rule’ appears to be a form of punitive disentitlement. As a recent case, *Henderson v Wilcox*, tells us, ‘There are two aspects of the rule to consider; the first is what crimes or acts are sufficient to engage the rule and the second is what rights or interests are affected by it’.^44^ It is more convenient to cover the latter aspect first. It tells us *how* an ‘offender’ is punished by telling us *from what* he could be disentitled.

The rule has extended to: (i) rights under insurance policies;^45^ (ii) rights of inheritance (under a will or intestacy law);^46^ (iii) rights under social security legislation (eg a widow’s pension);^47^ and (iv) rights of survivorship to co-owned property under a joint tenancy.^48^

There remains doctrinal uncertainty over the mode of disentitlement, and whether a uniform approach can or should be taken.^49^ A killer could be deprived through not obtaining any rights at all,^50^ a constructive trust,^51^ automatic severance of a joint tenancy^52^ or by being deemed to have died immediately before his victim (ie operation of a ‘predecease rule’).^53^

The forfeiture cases mostly involve the ‘preclusion’ or prevention of ‘profit’, rather than its divestment.^54^ The language of ‘forfeiture’ is therefore potentially misleading, suggesting that the killer must have already successfully acquired rights in or the value of a benefit, which are then subsequently stripped away. This is inaccurate. The examples demonstrate how property law and other private law rules could operate so that the killer never receives anything in the first place. Nothing is ‘transferred’,^55^ so there is nothing to restitute or disgorge. These are not cases of ‘restitution for wrongs’.

#### (ii) For what disentitled

The other aspect of the rule identified by Cooke J in *Henderson v Wilcox* tells us *for what* a person is being disentitled. What kind of conduct triggers forfeiture and what is the role of the killer’s fault—does the law take that into account? This generated controversy. Understanding the ‘forfeiture rule’ as punitive disentitlement is illuminating. It explains the importance of fault in determining whether such punishment is deserved.

The present law is not straightforward. An offender’s fault could feature at two stages:

1. Is the forfeiture rule engaged?
2. If engaged, should its legal effect (ie disentitlement) nevertheless be disapplied, partially or wholly, by a judge exercising their statutory discretion?

At stage 1, the positive law seems as follows: a criminal conviction is not necessary,^56^ but evidence of such is admissible in civil proceedings.^57^ Murderers are a core case.^58^ Even though it is now lawful to commit suicide, the Forfeiture Act catches persons who assist suicide, counting those who ‘aided, abetted, counselled or procured the death of that other’.^59^

But not all ‘unlawful killings’ count. It remains unclear whether all forms of manslaughter count.^60^ Part of this is because, as Salmon LJ helpfully reminds us in *Gray v Barr*, manslaughter is ‘a crime which varies infinitely in its seriousness. It may come very near to murder or amount to little more than inadvertence.’^61^ For similar reasons, until very recently it was unclear whether causing death by dangerous driving,^62^ or by careless or inconsiderate driving, counts.^63^ Suppose you are driving to the next town, with your father in the passenger’s seat. Unbeknownst to you, he has named you as sole heir in his will. You carelessly crash into a lamppost, causing your father’s death at the scene. Are you now forfeit from your inheritance? One might think that ought to depend on your culpability. Initially, however, the common law forfeiture rule seemed completely insensitive to fault. Moreover, it operated in an ‘all-or-nothing’ manner.^64^ Either you are disentitled or you are not. There was no middle ground.

A statute was passed to soften its impact: the Forfeiture Act 1982 granted the courts a discretionary power to make an order ‘modifying or excluding the effect of the rule’ if ‘the justice of the case requires’, but only for non-murder cases.^65^

The Act was enacted so that ‘criminal killing should not necessarily entail forfeiture and that there should be some proportionate relationship between the degree of moral guilt and the penalty which the perpetrator suffers’.^66^ The concern was with older cases like *Re Giles*,^67^ which had applied the forfeiture rule in a ‘draconian’,^68^ ‘rigid and some would say ruthless’^69^ fashion, without any regard to the culpability of the killer.^70^*Re Giles* was a ‘tragic’^71^ case of manslaughter by a battered wife with diminished responsibility. She was ultimately ordered to be detained in hospital for treatment of her mental illness.^72^ The judge notoriously held that ‘neither the deserving of punishment nor carrying a degree of moral culpability has ever been a necessary ingredient [for the operation of the forfeiture rule]’.^73^

Contrast cases decided after the Forfeiture Act 1982 came into force. *Re K*^74^ was another ‘tragic’^75^ case, involving yet another battered wife who, wielding a gun, had only intended to deter her husband from assaulting her after a trivial lunch incident. Instead, she accidentally shot him, killing him. Vinelott J exercised his discretionary power under the 1982 Act to disapply completely the forfeiture rule. He held that

cases of manslaughter necessarily vary infinitely in their gravity…. this is, I think, one of the cases which weighs least heavily…. She must accept the blame for what happened but she should not, in my judgment, suffer the further punishment of being deprived of the provision which her husband made for her.^76^

The forfeiture rule was again disapplied entirely in *Dunbar v Plant*, another ‘tragic’^77^ case, involving an unsuccessful suicide pact between young lovers. The fiancé died, but his fiancée survived with horrific injuries, despite repeated attempts to kill herself. The court held that while ‘The [statutory] discretion is a broad one … The first, and paramount consideration, must be whether the culpability attending the beneficiary’s criminal conduct was such as to justify the application of the forfeiture rule at all’.^78^ Revealingly, Phillips LJ observed that:

The rule is a judge-made rule to give effect to what was perceived as public policy at the time of its formulation. I believe that, but for the intervention of the legislature, the judges would themselves have modified the rule. Furthermore, it seems to me that the only logical way of modifying the rule would have been to have declined to apply it where the facts of the crime involved such a low degree of culpability, or such a high degree of mitigation, that the sanction of forfeiture, far from giving effect to the public interest, would have been contrary to it.^79^

Worries over whether application of the ‘forfeiture rule’ is deserved—focusing particularly on the killer’s culpability in causing the deceased’s death—demonstrates how it has been judicially and legislatively conceived as a form of *punitive* disentitlement. That is why it generated such controversy over the importance of fault. Punishing a faultless wrongdoer is problematic precisely because undeserved punishment still is punishment. Concerns of ‘double punishment’ where there has also been a criminal conviction reinforce the point.^80^

### B. Contractual Rights and Fiduciary Duties

What about contractual rights? There are cases, exemplified by *Imageview Management Ltd v Jack*,^81^ in which one party has been disentitled from their contractual rights to an agreed sum, which they have earned, and which would otherwise be due and payable by their counter-party. These seem instances of punitive disentitlement.

Within the agent–principal relationship, the main right of an agent against his principal is to remuneration for his services. This right is usually provided for by the express terms of an agency contract and described as a commission.^82^ However, an agent who commits a breach of duty—in particular, a fiduciary duty because of ‘an undisclosed but realistic possibility of a conflict of interest’^83^—has been said to ‘forfeit’ his contractual right to commission.

The leading case remains the Court of Appeal (CA) decision of *Imageview Management Ltd v Jack*.^84^ Kelvin Jack was a footballer from Trinidad and Tobago. He was goalkeeper for their national team. He wanted to play professionally in the UK, so he hired a sports agent, Imageview, to secure him a position at a UK football club, agreeing 10% of his monthly salary as the agent’s commission. Imageview successfully found him a two-year contract with Dundee Football Club. Everything proceeded smoothly for about a year, until it was discovered that, at the same time that Imageview was negotiating with Dundee Football Club on Jack’s behalf, they had also negotiated a secret side deal to receive £3000 from Dundee Football Club for obtaining Jack’s work permit. (The court found that the market value of such services would have been only about £750; when Jack inquired of the matter, Imageview told him ‘it was none of your business’.^85^)

Understandably, Jack refused to pay Imageview any outstanding commission. Imageview sued him for the remaining sum payable. Jack counter-claimed for a refund of commission paid, in addition to the £3000 secretly received by Imageview.

A unanimous CA held that Imageview lost. Jack won on all counter-claims:

[A]s the courts below held, there was a breach of fiduciary duty here. The cases I have cited make it plain that where there is such a breach commission is forfeit—so Mr Jack need pay no more agency fees and is entitled to repayment of the fees paid by him.^86^

By negotiating for a side deal in secret, the court thought there was a ‘clear’ conflict of interest because

it is possible that the more [Imageview] got for itself, the less there would or could be for Mr Jack. Moreover it gave Imageview an interest in Mr Jack signing for Dundee as opposed to some other club where no side deal for Imageview was possible.^87^

As Jacobs LJ explained:

The effect of [an agent’s] breach of duty was that he had to account for the commissions received and that he was not entitled to outstanding salary which otherwise would have been due. That clearly governs Imageview’s claim for outstanding commission which would otherwise have been due.^88^

The court also emphasised that ‘Once a conflict of interest is shown … the right to remuneration goes’.^89^ Thus, Imageview’s accrued contractual right to the agent’s commission, earned through providing Jack their club-finding services, was ‘forfeit’.

That such disentitlement was punitive is apparent from the judges’ reasoning and avowed aims. They sought to make an example of Imageview, demonstrating to all and sundry the harsh consequences befalling misbehaving agents who had ‘betrayed’ their principals.

Jacobs LJ regarded himself as being compelled not just by precedent, but also by ‘policy reasons’, because

if all the agent has to pay if and when he is found out are damages the temptation to betray the trust reposed in him is all the greater. So the strict rule is there as a real deterrent to betrayal.^90^

Agreeing with him Mummery LJ added:

In our age it is more important than it ever was for the courts to hold the precise and firm line drawn between payments openly, and therefore honestly, received by agents and undeclared payments received by agents secretly, and therefore justly liable to *all* the legal consequences flowing from breaches of an agent’s fiduciary obligations.^91^

The legal rule set out by *Imageview* is clear, but controversial.^92^ For some, it is controversial precisely *because* it is an instance of punitive disentitlement. The objection is that punishment is an inappropriate response; not all breaches of fiduciary duties are dishonest.^93^ Accordingly, a current editor of *Bowstead & Reynolds on Agency*,^94^ Peter Watts, has criticised the case for its ‘spectacularly punitive reasoning and result’,^95^ arguing that ‘To forfeit remuneration where the services that have been performed conform to the requirements of the contract of agency, as Imageview sanctions, is penal’.^96^

*Imageview* has been consistently applied by the lower courts, however.^97^ This has created a body of precedent so entrenched it can probably only be reviewed by the Supreme Court.^98^ In *Rahme v Smith*, Morgan J treated ‘the legal principles as to the consequences of a breach of fiduciary duty of this kind’ as ‘now well established’.^99^ In *Avrahami v Biran*, Newey LJ applied them to a dishonest fiduciary, forfeiting his contractual right to management fees in a joint venture project. He approved of the summary in *Snell’s Equity* that:

If a fiduciary acts dishonestly he will forfeit his right to fees paid or payable by the principal. He will also forfeit his right to such fees if he takes a secret profit from a third party which is directly related to performance of the duties in respect of which the fees were payable, even if the principal has benefited from the fiduciary’s performance of those duties.^100^

Most recently in *Staechelin v ACLBDD Holdings Ltd*, a case distinguishing *Imageview*, the CA reviewed the relevant principles and concluded unanimously that ‘dishonesty [is] the litmus test for forfeiture of commission’, so that ‘an agent will not lose his commission on account of a failure to pass on information if he has neither been dishonest nor acted in bad faith’.^101^ The CA emphasised how Jacobs LJ had said in *Imageview* that ‘A principal is entitled to have an honest agent, and it is only the honest agent who is entitled to any commission’.^102^

Under the present law, it therefore appears that an agent is being disentitled from his contractual rights to an agreed sum as a form of punishment for ‘dishonest’ breach of fiduciary duty, on some accounts an ‘equitable wrong’.^103^ The law punitively disentitles dishonest agents from their contractual rights to remuneration as a form of condemnation, in retribution for his ‘betrayal’ and ‘dishonesty’, for the explicit ‘policy’ reason of deterring him, and *pour discourager les autres*.^104^

### C. Unjust Enrichment and ‘Disqualifying Fault’

The law of unjust enrichment is especially debatable, but still worth mentioning. Suppose that, by mistake, C pays D £5000. As a result, D spends £4000 on a holiday she would not otherwise have taken. Can C claim restitution of the full £5000 from D?

Not if D is entitled to raise, in defence, her change of position—widely agreed to be the ‘most important’ and ‘characteristic defence in unjust enrichment’.^105^ It protects D by reducing *pro tanto* the quantum she may be compelled to repay to £1000.^106^ The defence was authoritatively recognised by the House of Lords in *Lipkin Gorman v Karpnale*.^107^ As Lord Goff said,

If the plaintiff pays money to the defendant under a mistake of fact, and the defendant then, acting in good faith, pays the money or part of it to charity, it is unjust to require the defendant to make restitution to the extent that he has so changed his position.^108^

#### (i) ‘Disqualifying fault’

Lord Burrows has recently said that change of position is ‘essentially an enrichment-based defence’.^109^ On such a view, a defendant is entitled to the protection of a change of position defence so long as she has disenriched herself in a relevant manner. The question is whether and how that protection can then be lost?^110^

Bant argued in her leading monograph on the topic that ‘the defendant’s conduct must not be such as to disentitle him from relying on the defence’, putting forth as one of its key rationales ‘restrict[ing] the level of protection given to the defendant by reference to the defendant’s fault … incorporat[ing] more overtly instrumentalist concerns’.^111^

Birks appears also to have conceived its operation as a form of disentitlement from the defence, coining the term ‘disqualifying fault’:

disqualification from the defence allows … liability to persist on the basis of the disqualifying fault … The defence ensures that the defendant, unless disqualified, will be strictly liable only to the extent that his assets remain swollen. Only a recipient who is disqualified will remain liable despite disenrichment, but for him the liability will not be strict, since on all views disqualification supposes fault on the part of the recipient.^112^

For our purposes, this feature stands out. It could potentially be understood as an example of punitive disentitlement, triggered by the presence of the extra facts of D’s misconduct in disenriching herself.

#### (ii) ‘Bad faith’, including dishonesty and sharp practice, and culpable ‘wrongdoers’

What counts as misconduct constituting ‘disqualifying fault’?

In *Lipkin Gorman*, Lord Goff said that ‘the defence is not open to one who has changed his position in bad faith … [or] … to a wrongdoer’.^113^ This sparked a chain of case law over the precise meaning and boundaries of ‘bad faith’ and ‘wrongdoer’.^114^

Stray cases have occasionally invoked the (less helpful) language of ‘unconscionable’ and ‘inequitable’.^115^ This might suggest to some that in deciding whether D is disentitled from the defence, a judge should balance the relative ‘equities’ of C and D,^116^to divide up discretionarily the ‘burden’, implementing some form of localised distributive justice.^117^ Such a view should and has been firmly rejected by the Privy Council in *Dextra Bank*, surveying the positions in the United States and in New Zealand.^118^ The defence would become ‘hopelessly unstable when it is used to reflect relative fault’.^119^ Moreover, it is inconsistent with well-established cases demonstrating that C’s negligence, D’s negligence and even D’s hardship are irrelevant.^120^ It is also inconsistent with the numerous landmark authorities emphasising repeatedly the priority of structured sequential legal rules over judicial discretion within this area of law.^121^

It is clear now that ‘bad faith’ does not include mere negligence,^122^ but that it does include ‘failure to act in a commercially acceptable way and sharp practice of a kind that falls short of outright dishonesty as well as dishonesty itself’.^123^

It is not far-fetched to think that a defendant might deserve punishment for their ‘dishonesty’. Perhaps even ‘sharp practice’, falling short of ‘outright dishonesty’, could make the cut. Consider a typical example of ‘bad faith’: *Cressman v Coys of Kensington*.^124^ That case involved a defendant who changed his position ‘with knowledge of the facts entitling the plaintiff to restitution’.^125^ A car purchaser, McDonald, was transferred title to a car as agreed under the contract of sale, but by mistake also a cherished personalised car registration mark.^126^ McDonald ‘knew that this was something that he was not supposed to have’,^127^ yet he tried to avoid restitution by gifting it to his partner, changing his position. Applying *Lipkin Gorman* and *Niru Battery*, Mance LJ disqualified him from the defence.^128^ McDonald’s gambit rightly failed.

### D. Highly Culpable Torts

Two examples will be raised from torts, part of the law of wrongs. As a precursor, it is useful to note that ‘fault’ and ‘wrong’ are distinct concepts within private law doctrine. Breach of a duty *owed to another* is a wrong done to them and only them. Some would therefore prefer the label ‘private wrong’, ‘civil wrong’ or ‘relational wrong’.^129^ Fault is a separate, orthogonal, matter.^130^

It is necessary, therefore, to distinguish between faulty wrongs and faultless wrongs. Private law recognises ‘strict’ duties, ie duties breachable *regardless of fault*. Breach of contract is by default a ‘strict’ wrong.^131^ Breach of trust is similarly strict. An innocent trespasser is an innocent (ie faultless) wrongdoer; there is no contradiction here.

#### (i) Contributory fault

The highly culpable conduct of a defendant could potentially disentitle him from raising contributory negligence. Widely treated as a ‘defence’,^132^ the apportionment legislation—the Law Reform (Contributory Negligence) Act 1945—now allows a reduction of damages by reference to the claimant’s contributory fault (ie a causally relevant failure to take reasonable care of themseves), ‘to such extent as the court thinks just and equitable’.^133^ Before that, it operated as a complete defence.

Despite being classed as a ‘general defence’ in *Clerk & Lindsell on Torts* alongside illegality and volenti,^134^ certain torts continue to be excluded from its scope. It is now clear from post-1945 authorities that the defence is inapplicable to torts like intentional trespass to goods,^135^ intentional trespass to the person,^136^ deceit,^137^ and other claims based upon dishonesty, ‘whether framed as fraud, conspiracy, inducing breach of contract, or whatever’.^138^

Why? Can its patchy scope be justified? Perhaps partially, if these rule-like exclusions or carve-outs from this ‘general’ defence—where the tortfeasor is dishonest or an intentional wrongdoer—are understood as instances of punitive disentitlement, motivated by and in response to the defendant’s culpable wrongdoing.^139^ One might explain contributory negligence in the standard cases where courts ‘balance’ the comparative fault of a careless defendant as against a careless claimant as a ‘device to effect loss-sharing’, instantiating localised distributive justice.^140^ But where ‘balancing’ or apportionment is not even considered, or thought offensive to consider, as in these blanket exclusions, the distributive explanation becomes unpersuasive, and requires supplementation.

Understanding these exclusions as the punitive disentitlement of intentional and fraudulent tortfeasors is a promising way of (at least partially) rationalising what is currently a bit of a chaotic mess. Here I borrow a suggestion from Glanville Williams in his seminal treatise *Joint Torts and Contributory Negligence*. Williams recognised that torts could be committed with varying levels of blameworthiness:

negligence is not only a tort in itself but also a way of committing other torts. Take, for instance, the tort of trespass to the person … the two modes in which the tort may be committed are therefore (1) the intentional and (2) the negligent. If on the facts the trespass was committed negligently, contributory negligence is as much a defence as if the action brought were one of negligence. Thus, to take an example, if an industrious student is locked in the library because he fails to observe the closing bell, his action of false imprisonment can be countered with the plea of contributory negligence if the defendant has not, on the facts, acted intentionally, even though false imprisonment as a tort can be committed intentionally.^141^

Importantly, Williams argued that ‘the exclusion of the defence in cases of intentional wrongdoing rests partly on ideas of policy; it is a penal provision aimed at repressing conduct flagrantly wrongful’.^142^

This justification was adopted by Aikens LJ in *Co-operative Group v Pritchard*, whose reasoning centred on ‘intentional torts’.^143^ As Goudkamp has helpfully reminded us, ‘batteries, for instance, may range from innocuous physical contact to brutal beatings, rape and murder. It follows that not all defendants who commit the tort of trespass to the person deserve punishment.’^144^

For another example of high culpability tortfeasors, consider deceit.^145^ Where the tort of deceit is made out, the fraudster is disentitled from raising his victim’s contributory negligence as a defence. As Lord Hoffmann observed in *Standard Chartered Bank v Pakistan National Shipping*, ‘This rule seems to me based upon sound policy. It would not seem just that a fraudulent defendant’s liability should be reduced on the grounds that, for whatever reason, the victim should not have made the payment which the defendant successfully induced him to make.^146^ (This coheres with the diminished causal requirement for contractual rescission against a fraudulent misrepresentor. As Lord Hoffmann pointed out, it is ‘a rule based upon moral disapproval of fraud’.^147^)

#### (ii) Remoteness

Rowan has demonstrated how, in French contract law, under article 1231-3 of the Civil Code, the contractual remoteness rule is disapplied where a breach is ‘grossly negligent’ (*faute lourde*) or deliberate or dishonest (*faute dolosive*), thus resulting in larger awards of damages. Reliance on exclusion and limitation clauses, and indemnification under an insurance policy, are not permitted. This seems a possible example of punitive disentitlement.^148^

Compare English tort law. A similar disapplication exists for fraudsters and intentional wrongdoers. On one view, a general remoteness doctrine protects tortfeasors from bearing an otherwise overwhelmingly burdensome duty to compensate tort victims for *all* losses suffered in consequence.^149^ Only reasonably foreseeable types of losses at the time of the tort count.^150^ Beyond that, a line is drawn, and the tortfeasor is protected.

However, since *Doyle v Olby*, there has existed a rule that a fraudulent tortfeasor is disentitled from that protection, so that all consequential losses are recoverable from him, regardless of their unforeseeability.^151^ As Lord Denning said, ‘All such damages can be recovered: and it does not lie in the mouth of the fraudulent person to say that they could not reasonably have been foreseen’.^152^ Examining that rule in *Smith New Court Securities v Citibank*, Lord Steyn explained its justification thus:

It may be said that logical symmetry and a policy of not punishing intentional wrongdoers by civil remedies favour a uniform rule. On the other hand, it is a rational and defensible strategy to impose wider liability on an intentional wrongdoer … The exclusion of heads of loss in the law of negligence, which reflects considerations of legal policy, does not necessarily avail the intentional wrongdoer. Such a policy of imposing more stringent remedies on an intentional wrongdoer serves two purposes. First it serves a deterrent purpose in discouraging fraud … in the battle against fraud civil remedies can play a useful and beneficial role. Secondly, as between the fraudster and the innocent party, moral considerations militate in favour of requiring the fraudster to bear the risk of misfortunes directly caused by his fraud.^153^

Lord Steyn’s reference to the fraudster having to bear the misfortune caused by his own fraud is supplementary, not alternative, to his goal of establishing a general ‘policy’ of ‘punishing intentional wrongdoers’ with ‘wider liability’ as ‘rational and defensible’.^154^

This deliberate carve-out looks like punitive disentitlement. It is an approach consistent and continuous with how, as above, tort law distinguishes between low-culpability wrongs and high-culpability wrongs, singling out only the latter as appropriate for punishment.^155^ Along similar lines, Goudkamp and Katsampouka have argued that ‘the courts, by relaxing the test for remoteness where the defendant has engaged in punishment-worthy behaviour, are, as they have made plain, acting retributively’.^156^ This judicial ‘dilution’ of the remoteness test is ‘animated’ by a ‘punitive impulse’ and generalisable to whenever the defendant intended to injure the claimant, or has acted dishonestly.^157^ It ranges across different torts, for example conspiracy,^158^ conversion,^159^ the statutory torts of discrimination^160^ and harassment.^161^

That the culpability of a wrongdoer might possess such significance is supported by Lord Blackburn’s less-well-remembered qualifier to his ‘general rule’ for tort damages in *Livingstone v Rawyard Coals Co*:

that, where any injury is to be compensated by damages, in settling the sum of money to be given for reparation of damages you should as nearly as possible get at that sum of money which will put the party who has been injured, or who has suffered, in the same position as he would have been in if he had not sustained the wrong for which he is now getting his compensation or reparation.^162^

But Lord Blackburn immediately goes on to describe how:

That must be qualified by a great many things which may arise—such, for instance, as by the consideration whether the damage has been maliciously done, or whether it has been done with full knowledge that the person doing it was doing wrong. There could be no doubt that there you would say that everything would be taken into view that would go most against the wilful wrongdoer—*many things which you would properly allow in favour of an innocent mistaken trespasser would be disallowed as against a wilful and intentional trespasser* on the ground that he must not qualify his own wrong, and various things of that sort.^163^

Lord Blackburn’s reference to ‘disallowing’ what would be ‘properly allowed in favour of’ low culpability tortfeasors describes well a judicial practice of disentitling high culpability tortfeasors from remoteness doctrines on punitive grounds.

## 5. Implications

If am I right in identifying this phenomenon, so what? It may help to pull together threads from the preceding discussion first.

### A. Summary

As a wide-ranging ‘principle of public policy’, the ‘forfeiture rule’ has been judicially, and legislatively, conceived as a form of punitive disentitlement for ‘unlawful killing’. Hence the controversy generated over its operation without due regard to the culpability of persons causing death. This ‘principle of public policy’ disqualified them from rights under insurance policies, rights of inheritance, rights under social security legislation (eg a widow’s pension) and rights of survivorship.

In what has been criticised as ‘spectacularly punitive’, accrued contractual rights to agreed sums have been ‘forfeited’ from agents who breached their fiduciary duties, in retribution for ‘betraying’ the trust reposed in them by their principals. They are condemned in ‘emphatic terms’.^164^ Said censure is also coupled with a ‘policy’ aimed at deterring dishonest agents and discouraging others from misbehaving similarly.

When restitution for an unjust enrichment is sought, defendants guilty of ‘disqualifying fault’ are disentitled from raising their change of position in defence, such as those who have disenriched themselves ‘dishonestly’. Courts have noted that ‘bad faith’ includes ‘dishonesty’ and ‘sharp practice’.

Intentional and fraudulent tortfeasors are completely disentitled from raising the defence of contributory negligence, which would otherwise reduce damages based on the claimant’s contributory fault. Similarly, highly culpable tortfeasors, such as those who have intended to injure the claimant, or who have acted dishonestly, have been disentitled from the protection of a remoteness doctrine for ‘moral considerations’ and to ‘serve a deterrent purpose’.

I hope in the preceding sections to have identified enough potential examples of punitive disentitlement, from across a sufficiently broad range of doctrines across different categories of private law, to have successfully challenged what appears a widespread assumption: that punitive damages—which has occupied centrestage as our paradigm example of punishment within private law—is the *only* form of punishment relevant to private lawyers, the legitimacy of which is worth debating. Punishment could take multiple forms. Disentitlement too could be punitively motivated, and, especially if punishment is thought ‘anomalous’^165^ or problematic within private law’s structure, that too is worth our attention and reflection.

The point was not to provide an exhaustive list or taxonomy of all possible instances of punitive disentitlement. Reasons of space prevent that, though this article will have hopefully paved the way for further work along these lines. The much more modest aim was simply to demonstrate that the problem of punishment in private law may be more far-reaching than conventionally thought or assumed. There are instances more diffuse, and more unsystematic, which have been less well recognised and interrogated. The only way to bring greater attention to the phenomenon was to collect together separate instances in a broad, necessarily brief, survey of plausible examples from within private law doctrine. This was done by considering a separate but less obvious form of punishment—punishment through disentitlement—wherein a person is disentitled from a legal right, defence or other legal advantage they would or should otherwise be entitled to, in response to and because of some alleged or supposed misconduct on their part.

### B. Justifiability

Rawls once suggested that we could reconcile retributive versus utilitarian views of punishment, two views said to be in tension, ‘by the time-honoured device of making them apply to different situations’. So utilitarian arguments about punishment (by reference only to consequences) are appropriate to questions about practices, justifying what the legislator does *qua* legislator. But retributive arguments fit the application of particular rules to particular cases, justifying what the judge does *qua* judge.^166^The latter seems more applicable to private law doctrine, the bulk of which has evolved casuistically through common law adjudication in adversarial proceedings between particular litigants.

Should we continue punishing through disentitlement in private law? We might begin by asking if punishment through disentitlement is open also to the same normative objections as punitive damages.

#### (i) Advantages

Numerous questions have been raised about the legitimacy of using private law damages awards as a means of punishing a defendant for his civil wrong (ie a tort, breach of contract, or equitable wrong). The objections have been so well rehearsed that it will be of limited utility repeating them in detail here.^167^ Unsurprisingly, some of these objections apply with similar force to punitive disentitlement, for example, the lack of procedural safeguards provided by criminal proceedings.^168^

Some, however, may not. A key normative objection of abolitionists is that punitive damages are incompatible with a private law concerned with interpersonal justice between claimant and defendant, and that is fundamentally bilateral in structure. Deterrence focuses on its future effect on public behaviour, treating damages not as a remedy for a wrong that the defendant did to the claimant, but instead as a policy lever to provide incentives or disincentives to parties not before the court. Condemnation or censure focuses only on the defendant’s past conduct, in particular his culpability and desert.^169^

These aims of punishing a defendant cannot explain or justify the claimant’s role and his connection to the defendant, except perhaps only contingently. It cannot justify why only the victim should have the standing to hold the defendant accountable, or why the claimant is entitled to receive damages quantified by reference to defendant-centric or public-facing aims. Unlike deterrence and condemnation, retribution is the only punitive aim that could potentially connect the defendant’s wrongdoing to a particular claimant in a non-contingent way.^170^

As Lebel J (dissenting) commented in *Whiten*, ‘By reason of the relational nature of private tort law, punitive damages do not fit easily into its overall scheme … Punitive damages differ strikingly from all other damages as the sole reason for awarding them is to punish’.^171^

Thus, a central, and to my mind powerful, normative objection to punitive damages is that it entails illegitimately giving someone a power to enforce a duty of the defendant that is not owed to them (but rather owed to the public perhaps), coupled with a right to receive damages that are a ‘pure and undeserved windfall at the expense of the defendant’.^172^ As Lord Reid explained in *Cassell v Broome:*

courts, perhaps without fully realising what they were doing, appeared to have permitted damages to be measured not by what the plaintiff was fairly entitled to receive but by what the defendant ought to be made to pay as punishment for his outrageous conduct.^173^

Articulating a similar worry, Penner argued that:

One cannot derive a right to punish, which … is a collective, public right, from a private right … Nor can the private rights of the plaintiff generate any right to the benefit of a punishment … As a public measure of condemnation, a punishment cannot allocate the benefit of a punishment to any private actor. In that respect punitive damages privatise punishment, and that alone brings them worryingly close to a form of private retaliation. But it gets worse … the power to enter into a settlement … brings the whole matter as close as can be to state-backed private retaliation for personal gain.^174^

The illegitimate conferral of legal entitlements to private individuals, entailed by an award of punitive damages, is absent in *dis*entitlement, for it does the opposite. No one is given anything. Instead, someone is precluded from something. Rightly, therefore, the spotlight is on the person being disentitled, and whether he deserved it in light of his misconduct. The focus is, and should be, unilateral rather than bilateral.

At first glance, punitive disentitlement appears less objectionable than punitive damages. However, there are problems uniquely associated with punitive disentitlement that counsel caution against its continued or widespread use.

#### (ii) Concerns

Is the practice consistent with principles that ought to govern punishment more generally? All forms of punishment, including punitive disentitlement, should be subject to moral constraints. A just system of punishment at the very least requires that (i) the hard treatment inflicted on the ‘offender’ bears some reasonable relation to the gravity of their misconduct (sometimes phrased in terms of ‘proportionality’) and that (ii) only conduct sufficiently blameworthy or culpable is punishable (‘moral desert’).^175^

A potential objection to the practice of punitive disentitlement is that it may not meet these moral constraints. Principally, this is due to its relative lack of scalability as compared to punitive damages, the criminal analogue of which are fines. Like discretion in sentencing, it has been argued that a court order of punitive damages can be judicially calibrated ‘by reference to the defendant’s desert’.^176^ The relative lack of flexibility and judicial discretion in what I have called disentitlement has been said to render it a ‘decidedly inferior way of achieving retributive justice relative to punitive damages’, as it raises a potential for ‘disproportionate punishment’.^177^

The lack of scalability in punitive disentitlement is due to two reasons. The first is that the maximum burden imposable through disentitlement is capped at the maximum value of the entitlement in question, for example a contractual right to be paid a £100,000 debt.^178^ The second is that there may be no viable option to adjust downwards the extent of hardship inflicted, by partial disentitlement only. As discussed above, judges possess a statutory discretion to disentitle an ‘unlawful killer’, say, of only half of their inheritance rather than all of it, depending on the ‘justice of the case’.^179^ Depending on the legal doctrines involved, however, it is not always easy to ensure that punitive disentitlement does not become wholly out of proportion to the gravity of the misconduct.

As an illustration, take an instance where it has been thought that such a difficulty might be encountered. If dishonest tortfeasors are punished by disentitling them from defences otherwise applicable—say, for instance, contributory negligence or the remoteness doctrine—their ultimate burden borne in the form of compensatory damages payable to their victims could become wholly arbitrary, and even potentially extreme.^180^ This is because the losses a victim suffers as a consequence of a tort can depend on luck, such as the possibility of intervening events which could aggravate the loss, or the especial vulnerability of the victim, like his ‘thin skull’ or his especially large earning potential.^181^

We do not, however, normally think it is morally permissible for the severity of the punishment to depend on chance. That would be a form of ‘penal lottery’—a system of punishment in which the offender is subjected to a risk of punitive burden, such that if he wins the lottery, he escapes the burden, but if he loses, he does not.^182^

There is truth to these concerns. They demand that we be more alert to, and wary of, the practice of punitive disentitlement. In each case, it must be asked whether disentitling someone as a means of punishing them is compatible with the moral constraints on legitimate punishment more generally.

The difficulties should not be overstated, however. First, they are much ameliorated if the decision about whether to disentitle takes place at time of trial. By then, many facts and events, such as the extent of consequential losses suffered by the claimant, will be known.^183^ A judge could then consider whether disentitling a tortfeasor from the protection of a defence he should otherwise be entitled to raise, on grounds that he ought to be punished for, say, his dishonest or fraudulent conduct, would constitute disproportionate punishment.^184^ The main question is whether the legal rules implicated allow for such adjustment; this seems an issue at the level of rule design rather than rule application.^185^ If the rule is a purely common law rule, such as remoteness or change of position, there is latitude for judicial rule modification to accommodate this concern; less so if statutory intervention has been applied atop the common law inhibiting its judicial development, as in the case of contributory negligence.

Second, and more importantly, the danger will not arise in all cases, so we should not be too quick to generalise. It will not exist, for instance, in a claim for restitution of a mistaken payment of, say, £50,000. The nature of the claim sets a natural cap on the maximum quantum a defendant might ever be compelled to repay (ie £50,000), even if, after having disenriched herself, she were disentitled from raising her change of position as a defence. It should be apparent that difficulties in calibration depend on at least two matters: first, the nature of the entitlement potentially at stake (ie whether it is a legal right, defence, or some other legal advantage); and second, the existence and nature of any claims in the background (ie whether one is subject to action for an agreed sum, compensatory damages for a tort or breach of contract, restitution for unjust enrichment, or an account of profits for breach of fiduciary duty, etc).

Third, difficulties in scaling are anyway faced by all sorts of non-financial punishments, for example probations or curfews, incarceration, smacks on the wrists, caning, or even death. Typically, a person has only two wrists, one body, and one life; these cannot be apportioned into infinitely divisible parts for calibration. It is only sanctions like fines, because they are financial, that lend themselves to gradation at a granular level. Even then, there are limitations. Their scalability depends on the wealth—or, conversely, the poverty—of the defendant. The bankrupt are judgment-proof and the wealthy substantively unhurtable through financial sanctions alone.^186^ It does not seem plausible that we should, on grounds of scalability, abolish all other sanctions and confine criminal sanctions exclusively to fines. That would be a charter for the rich to go rogue. For crimes inflicting physical suffering on others, possibly even death, a purely financial punishment seems wholly insufficient. Some retributivists might say that the punishment must resemble the offence committed in kind and in degree; or at least, the response cannot be of an entirely different kind.

## 6. Conclusion

Whatever its form, in whichever area of law, to be acceptable punishment must satisfy moral constraints. Punitive disentitlement may have flown under the radar. We should be more alert to, and concerned about, its coherence and justifiability.

Doubtless, some will remain unconvinced. They may be committed to a specific, different conception of ‘punishment’. They may profess different justifications. They may contend that our legal entitlements ought to be of a different structure or scope. Many of the doctrines discussed are controversial. Private lawyers continue to dispute how they ought best be understood and justified. I have proposed but one interpretation, explaining why it might be plausible.

We might stipulate (revisionary) definitions. We might say these are mere ‘penalties’, and not ‘punishments’. That is not a resolution. Even to ‘substandard or secondary’ instances of punishment, constraints must apply.^187^ Whether ‘punitive’ or ‘penal’ disentitlement, to the disentitled it may be no less unjust or undeserved. As Feinberg said:

It is useful to distinguish violations and civil penalties from crimes and punishments; but it does not follow that the safeguards of culpability requirements and due process which justice demands for the latter are always irrelevant encumbrances to the former. Two things are morally wrong: (1) to condemn a faultless man while inflicting pain or deprivation on him however slight (unjust punishment); and (2) to inflict unnecessary and severe suffering on a faultless man even in the absence of condemnation (unjust civil penalty).^188^

## Footnotes

1 See eg A. Mitchell Polinsky and Steven Shavell, ‘Punitive Damages: An Economic Analysis’ (1998) 111 Harv L Rev 869; Keith Hylton, ‘Punitive Damages and the Economic Theory of Penalties’ (1998) 87 Georgetown Law Journal; Ralph Cunnington, ‘Should Punitive Damages Be Part of the Judicial Arsenal in Contract Cases?’ (2006) 26 LS 369; James Edelman, ‘In Defence of Exemplary Damages’ in Rickett (ed), *Justifying Private Law Remedies* (Hart Publishing 2008); Solène Rowan, ‘Reflections on the Introduction of Punitive Damages for Breach of Contract’ (2010) 3 OJLS 495; James Goudkamp and Eleni Katsampouka, ‘Punitive Damages and the Place of Punishment in Private Law’ (2021) 84 MLR 1257; James Goudkamp and Eleni Katsampouka (eds), *Landmark Cases in the Law of Punitive Damages*, (Hart Publishing 2023); Elise Bant and others (eds), *Punishment and Private Law* (Hart Publishing 2021): an entire part dedicated to ‘Punitive Damages’.

2 Allan Beever, ‘The Structure of Aggravated and Exemplary Damages’ (2003) 23 OJLS 87, 105–10; Ernest Weinrib, *Corrective Justice* (OUP 2012) 170–1: ‘punitive damages … are encased in controversy … That corrective justice renders punitive damages problematic is obvious on its face’; cf Robert Stevens, *Torts & Rights* (OUP 2007) 85, who re-explains punitive damages ‘as a form of substitutive damages for the right infringed’, aimed at retribution rather than deterrence; Arthur Ripstein, *Private Wrongs* (Harvard UP 2016) 260–1 similarly re-explains them as either gain-based or aggravated damages, arguing that ‘The practice of the US courts with regard to punitive damages gives up on the idea that a tort is a private wrong’; James Penner, ‘Punishments and Penalties in Private Law, with Particular Reference to the Law Governing Fiduciaries’ in Bant and others (n 1) 117.

3 See eg Andrew Burrows, *Remedies for Torts, Breach of Contract, and Equitable Wrongs* (4th edn, OUP 2019) 375–8. See also Law Commission, *Aggravated, Exemplary and Restitutionary Damages* (Law Com No 247, 1997), Parts IV and V; Cunnington (n 1); Goudkamp and Katsampouka, ‘Place of Punishment’ (n 1).

4 HLA Hart, *Punishment and Responsibility* (John Gardner ed, 2nd edn, OUP 2008) xlix–l.

5 (1990) 12 Cr App R (S) 262, 267.

6 Nigel Walker, *Why Punish?* (OUP 1991) 1.

7 Gabriel Chin, ‘The New Civil Death: Rethinking Punishment in the Era of Mass Conviction’ (2012) 160 U Pa L Rev 1789, 1790.

8 See eg Andrew Von Hirsch and Martin Wasik, ‘Civil Disqualifications Attending Conviction: A Suggested Conceptual Framework’ (1997) 56 CLJ 599, 601–4; Mirjan Damaska, ‘Adverse Legal Consequences of Conviction and Their Removal: A Comparative Survey’ (1968) 59 Journal of Criminal Law, Criminology and Police Science 347; TR McCoy et al, ‘The Collateral Consequences of a Criminal Conviction’ (1970) 23 Vand L Rev 929.

9 Representation of the People Act 1983, s 3.

10 Road Traffic Offenders Act, ss 34–43.

11 Licensed Premises (Exclusion of Certain Persons) Act 1980, s 1.

12 Football Spectators Act 1989 Part II, as amended by Football (Disorder) Act 2000.

13 Animal Welfare Act 2006, ss 33–5.

14 Jeremy Travis, ‘Invisible Punishment: An Instrument of Social Exclusion’ in Mauer and Chesney-Lind (eds), *Invisible Punishment: the Collateral Consequences of Mass Imprisonment* (The New Press 2003); Jeff Manza and Christopher Uggen, *Locked Out: Felon Disenfranchisement and American Democracy* (OUP 2006); James Jacob, *The Eternal Criminal Record* (Harvard UP 2015); Hugh Lafollette, ‘Collateral Consequences of Punishment: Civil Penalties Accompanying Formal Punishment’ (2005) 22 Journal of Applied Philosophy 241.

15 Parliamentary Elections Act 1954 (Singapore), s 5: ‘Qualification of electors’.

16 Parliamentary Elections Act 1954 (Singapore), s 6: ‘Disqualification of electors’.

17 Non-punitive disentitlements could include eg rights terminating on the debtor’s insolvency (which might offend the anti-deprivation principle): *British Eagle International Airlines v Cie Nationale Air France* [1975] 1 WLR 758 (HL); *Belmont Park Investments Pty Ltd* v *BNY Corporate Trustee Services Ltd* [2011] UKSC 38, [2012] 1 AC 383; or the various restrictions on undischarged bankrupts: Insolvency Act 1986, ss 31, 360, 390, 426A, 426B; Charities Act 2011, s 178; Local Government Act 1972, s 80; Company Directors Disqualification Act 1986, s 11; Pensions Act 1995, s 29.

18 See eg the controversy generated by tennis superstar Novak Djokovic in 2022, which made world news headlines: *Djokovic v Minister for Immigration, Citizenship, Migrant Services and Multicultural Affairs* [2022] FCAFC 3 <www.nytimes.com/2022/01/05/sports/tennis/novak-djokovic-australia-visa.html>.

19 Compare John Gardner, ‘Wrongs and Faults’ in AP Simester (ed), *Appraising Strict Liability* (OUP 2005) 71–4: ‘The fault principle is a principle governing punishment, and it applies to the criminal law because criminal liability is a liability to be punished … a punishment … is *deserved* only if the wrong being punished was faultily committed … Punishing a faultless wrongdoer is punishing undeservedly. But it is still punishing’ (emphasis in original).

20 see eg Thomas Hobbes, *Leviathan* (first published 1651; Tuck ed, CUP 1996) ch xxviii; Jeremy Bentham, *The Principles of Morals and Legislation* (London 1780) ch xii, para 36 and ch xv, para 28; Jeremy Bentham, *The Rationale of Punishment* (London 1830) bk 1, ch 1; Antony Flew, ‘The Justification of Punishment’ (1954) 29 Philosophy 291; John Rawls, ‘Two Concepts of Rules’ (1955) 64 Philosophical Review 3, 10; SI Benn, ‘An Approach to the Problems of Punishment’ (1958) 33 Philosophy 325; Joel Feinberg, ‘The Expressive Function of Punishment’ (1965) 49(3) Monist 391; Uma Narayan, ‘Appropriate Responses and Preventive Benefits: Justifying Censure and Hard Treatment in Legal Punishment’ (1993) 13 OJLS 166; Arthur Ripstein, *Equality, Responsibility and the Law* (CUP 1999) 140; Kit Barker, ‘Punishment in Private Law—No Such Thing (Any More)’ in Bant and others (n 1).

21 Flew (n 20) 291; Nicola Lacey, *State Punishment* (Routledge 1988) 6: ‘it would be naïve to expect even a detailed definition [of punishment] (comprised itself of open-textured language) to conclude all questions about the meaning of the word’.

22 HLA Hart, *Punishment and Responsibility* (n 4) 4–5.

23 As shall be seen below in section 4, disentitlement could sometimes occur ‘automatically’ by operation of law (ie the legal rules), rather than necessarily through a ‘judicial act’, ie a court ruling or order as in punitive damages awards. The relevant question is hence whether the *legal rules* with disentitling effect are operating as intended. Outside the punitive damages paradigm, the distinction between ‘punitive in effect’ and ‘punitive in intent (or aim)’ is not easy, and may obscure more than it reveals. I am presently inclined to think that ‘intended at’ includes oblique intention, ie treating as intended an effect foreseen as virtually certain to occur as a result of inflicting hard treatment. So, for example, I am sceptical about judicial pronouncements that accounts of profits in equity are not ‘punitive’ simply because they are supposedly not punitive in ‘intent’ or ‘aim’, but only punitive in ‘effect’: *Vyse v Foster* (1872) LR 8 Ch App 309, 333 (James LJ); *Harris v Digital Pulse Pty Ltd* [2003] NSWCA 10 [171], [191]–[213] (Mason P (dissenting)); James Edelman, ‘A “Fusion Fallacy” Fallacy?’ (2003) 119 LQR 375; *Attorney-General v Blake* [2001] 1 AC 268, 295 (Hobhouse (dissenting)): ‘essentially punitive nature of the claim’. Compare also Elise Bant and Jeannie Paterson, ‘Effecting Deterrence through Proportionate Punishment’ in Bant and others (n 1) 256–7, 269, 274, 276–85, arguing that courts do exercise their equitable and ‘civil penalties’ jurisdictions to punish (proportionately) in order to deter. See also n 26. Thanks to the anonymous reviewers for prompting me to reflect on these matters.

24 Lacey (n 21) 4, 7–12.

25 While there are many varieties of ‘expressivism’, see eg Andrew von Hirsch, *Censure and Sanctions* (OUP 1993), the classic example remains Feinberg (n 20), for whom ‘Punishment is a conventional device for the expression of attitudes of resentment and indignation, and of judgments of disapproval and reprobation’. cf HLA Hart, *Law Liberty and Morality* (OUP 1963) esp 65: ‘Is the mere expression of moral condemnation a thing of value in itself to be pursued at this cost? The idea that we may punish offenders against a moral code, not to prevent harm or suffering or even the repetition of the offence but simply as a means of venting or emphatically expressing moral condemnation, is uncomfortably close to human sacrifice as an expression of religious worship … The normal way in which moral condemnation is expressed is by *words* … Why should a denunciation take the form of punishment?’ (emphasis original) cf also n 26.

26 Barker (n 20) argues that the ‘*meaning*’ of ‘punishment’ varies according to judicial aims, and that many different possible aims are possible: (i) retributive justice; (ii) corrective justice; (iii) distributive justice; (iv) protection, prevention (prophylaxis) and rehabilitation; (v) deterrence; (vi) appeasement through catharsis; and (vii) social messaging. Through conceptual (rather than normative or evaluative) analysis, Barker argues we should limit ‘punishment’ to the singular ‘core’ aim of ‘retributive justice or reprisal’, which he argues does not exist in private law doctrine. Hence his conclusion that ‘there is no such thing’ as ‘punishment in private law’ (as he defines it). Note also that because Barker thinks ‘we … are concerned with judicial orders and their purposes, not their effects’ (42–4), he ends up having to discard the ‘hard treatment’ requirement widely thought necessary (criterion (a)): on which see eg Narayan (n 20). I remain sceptical about this move (under this view even a damages award of £1 could qualify as ‘punitive’, so long as ‘retributory’ in ‘aim’). I am also sceptical about the viability of his aims/effects distinction (n 23). Some of the judicial ‘aims’ Barker identifies overlap, and, as he notes, are not easy to ascertain (43).

27 (2002) 209 DLR (4th) 257, [2002] 1 SCR 595 [94]. cf *Cassell v Broome* [1972] AC 1027 (HL) 1073 (Lord Hailsham); *Rookes v Barnard* [1964] AC 1129 (HL) 1228 (Lord Devlin).

28 [2015] UKSC 67, [2016] AC 1172 [243] (Lord Hodge).

29 [2015] UKSC 67, [2016] AC 1172 [32], [82] (Lords Neuberger, Sumption, and Carnwath): ‘[some clauses] may be described as a deterrent … that is only objectionable if … the object was to punish’. cf *Dunlop Pneumatic Tyres Co v New Garage and Motor Co* [1915] AC 79 (HL) 86 (Lord Dunedin).

30 See further n 2; text to nn 167–174.

31 Hart (n 22) 5–6: ‘No account of punishment can afford to dismiss this question with a definition’. Hart calls this abuse the ‘definitional stop.’ See also Lacey (n 21) 6, 12–15.

32 Forfeiture Act 1870, 33 & 34 Vict c23; *Beresford v Royal Insurance* [1937] 2 KB 197 (CA), 211 (Lord Wright MR). See also S Bridge, E Cooke and M Dixon (eds), *Megarry and Wade Law of Real Property* (9th edn, Sweet & Maxwell 2019) [20-10], [20-22]; Roger Kerridge, ‘Visiting the Sins of the Fathers on Their Children’ (2001) 117 LQR 371, 374; A Reppy, ‘The Slayer’s Bounty—History of the Problem in Anglo-American Law’ (1942) 19 NYU LQ Rev 229.

33 See n 39.

34 [1892] 1 QB 147 (CA).

35 Under the Married Women’s Property Act 1882, s 11.

36
*Cleaver* (n 34) 158 (Fry LJ), 154: ‘treated as if it did not exist’ (Lord Esher MR), 160 ‘regarded as struck out’ (Lopes LJ).

37 ibid 156.

38 ibid 156.

39 See eg *Re Crippen* [1911] P 108, 112; *Re Hall* [1914] P 1, 5; *Re Sigsworth* [1935] Ch 89; *Re Callaway* [1956] 1 Ch 559, 562; *Re Giles* [1972] Ch 544, 551; *Re Royse* [1985] Ch 22; *Re K* [1985] Ch 85.

40 (n 39) 112 (Sir Samuel Evans, President).

41 (n 39) 8 (Hamilton LJ).

42 (n 39) 552 (Pennycuick VC).

43 Forfeiture Act 1982, s 1(1).

44 [2015] EWHC 3469 (Ch), [2016] 4 WLR 14 [9] (David Cooke J).

45
*Cleaver* (n 34); *Beresford v Royal Insurance Co Ltd* [1938] AC 586 (HL); *Dunbar v Plant* [1998] Ch 412.

46
*Hall* (n 39) (will); *Crippen* (n 39) (intestacy); *Re Sigsworth* (n 39) (will or intestacy); *Henderson v Wilcox* [2015] EWHC 3469 (Ch), [2016] 4 WLR 14 (will); *Henderson v Dorset Healthcare University NHS Foundation Trust* [2020] UKSC 43, [2020] 3 WLR 1124. NB an estate-beneficiary obtains no rights to particular assets before administration, only a right to due administration: *Commissioner of Stamp Duties v Livingston* [1965] AC 694 (PC), [1964] 3 WLR 963.

47
*R v Chief National Insurance Commissioner* [1981] QB 758.

48
*Re K* (n 39) (Vinelott J), decision affirmed [1986] Ch 180 (CA).

49 Roger Kerridge, *The Law of Succession* (13th edn, Sweet & Maxwell 2016) [14-64]; Ian Williams, ‘How Does the Common Law Forfeiture Rule Work?’ in Haecker and Mitchell (eds), *Current Issues in Succession Law* (Hart Publishing 2016).

50
*Re K* (n 39), discussed Williams (n 49) 63–4: ‘*Re K* suggests that a killer never acquires any rights in relation to the estate, not even the right to its due administration.’

51 James Barr Ames, ‘Can a Murderer Acquire Title by his Crime and Keep it?’ (1897) 45 American Law Register and Review 225.

52
*Dunbar v Plant* (n 45).

53 Estates of Deceased Persons (Forfeiture Rule and Law of Succession) Act 2011. Inserting Wills Act 1837, s 33A and Administration of Estates Act 1925, s 46A after *Re DWS* [2001] Ch 568 (CA). See also Law Commission, ‘The Forfeiture Rule and the Law of Succession’ (Law Com No 295). Traceable to AW Scott, *Scott on Trusts* (4th edn, 1988) para 492, adopted in the American Restatement of Restitution under the heading ‘Constructive Trusts’.

54 See n 43. See eg *R v Chief National Insurance Commissioner* (n 47); *Glover v Staffordshire Police Authority* [2006] EWHC 2414 (admin); *Re Pollock* [1941] Ch 219 (Ch); *Crippen* (n 39); *Re K* (n 39); Williams (n 49) 62: ‘The dominant view in England is that in some sense the unlawful killer does not obtain rights to assets. Quite what this means, and how this occurs, is much less clear’.

55
*ITC v HMRC* [2017] UKSC 29 [42]–[43].

56
*Gray v Barr* [1971] 2 QB 554 (CA); *Dunbar v Plant* (n 45). A Learmonth and others (eds), *Williams, Mortimer & Sunnucks on Executors, Administrations and Probate* (22nd edn, Sweet & Maxwell 2023) [64-02].

57
*Crippen* (n 39); Civil Evidence Act 1968, s 11.

58
*Cleaver* (n 34); *Crippen* (n 39), *Re Sigsworth* (n 39); *Re Pollock* (n 54); *Re DWS* (n 53).

59 Forfeiture Act 1982, s 1(2); Suicide Act 1961, ss 1, 2, 2A, 2B; *Ninian v Findley* [2019] EWHC 297 (Ch) (wife helped over-80 husband diagnosed with incurable progressive supranuclear palsy commit suicide in Switzerland at the Dignitas clinic).

60
*Hall* (n 39); *Re Dellow’s WT* [1964] 1 All RT 771; *Re Giles* (n 39); *Henderson v Wilcox* (n 46). cf *Dunbar v Plant* (n 45) 421–6 (Mummery LJ), 435 (Phillips LJ).

61
*Gray v Barr* (n 56) 581 (Salmon LJ).

62 Road Traffic Act 1988, s 1.

63 Road Safety Act 2006, s 20; *Amos (Deceased) Re* [2020] EWHC 1063 (Ch), [2020] 4 WLR 86.

64 Stephen Cretney, ‘The Forfeiture Act 1982: The Private Member’s Bill as an Instrument of Law Reform’ (1990) 10 OJLS 289, 294.

65 Sections 2, 2(2), 5.

66 For background see Cretney (n 64) 300. For criticism, see Paul Matthews, ‘Property, Pensions and Double Punishment: The Forfeiture Act 1982’ (1983) 5 JSWL 141; Phillip Kenny, ‘Forfeiture Act 1982’ (1983) 46 MLR 66; Kerridge, *The Law of Succession* (n 49) [14-66].

67
*Re Giles* (n 39). TK Earnshaw and PJ Pace, ‘“Let the Hand Receiving It be ever so Chaste …”’ (1974) 37 MLR 481.

68 Cretney (n 64) 289.

69 Leo Abse MP, Report of House of Commons Standing Committee C, 17 March 1982, cols 1 and 2.

70 Similarly, *R v Chief National Insurance Commissioner* (n 47).

71
*Re Giles* (n 39) 553.

72 Under the Homicide Act 1957, s 2 and the Mental Health Act 1959, s 60.

73
*Re Giles* (n 39) 552 (Pennycuick VC), citing *Hall* (n 39) 7 (Hamilton J).

74
*Re K* (n 39), affirmed [1986] Ch 180 (CA). Cretney (n 64) 302: ‘what court on these facts would consider a fine of almost half a million pounds to be an appropriate response to the widow’s culpability?’

75
*Re K* (n 39) 102.

76 ibid 102.

77
*Dunbar v Plant* (n 45) 438.

78 ibid 438.

79 ibid 435.

80 See eg the less-cited dissent in the case popularised by Ronald Dworkin, *Riggs v Palmer* (1889) 115 NY 506, 519 (Gray J): ‘to concede appellants’ views would involve the imposition of an additional punishment or penalty upon the respondent … The law has punished him for his crime, and we may not say that it was an insufficient punishment’. cf WB Meyer, ‘The Background to Riggs v. Palmer’ (2020) 60 Am J Legal Hist 48.

81 [2009] EWCA Civ 63, [2009] 1 Lloyd’s Rep 436. Restating and consolidating previous authorities: *Boston Deep Sea Fishing v Ansell* (1888) 39 Ch D 330 (CA); *Andrews v Ramsay* [1903] 2 KB 635; *Rhodes v Macalister* (1923) 29 Com Cas 19 (HC); *Hippisley v Knee Bros* [1905] 1 KB 1; *Keppel v Wheeler* [1927] 1 KB 57.

82 Peter Watts, ‘Forfeiture of Agents’ Remuneration’ in Devonshire and Havelock (eds), *The Impact of Equity and Restitution in Commerce* (Hart Publishing 2019) 203.

83
*Imageview* (n 81) [6].

84 ibid.

85 ibid [2].

86 ibid [51].

87 ibid [5].

88 ibid [14].

89 ibid [44].

90 ibid [50].

91 ibid [65] (original emphasis).

92 Watts (n 82); Peter Watts, ‘Restitution and Conflicted Agents’ (2009) 125 LQR 369; Seb Oram, ‘Forfeiture of Fiduciary Remuneration Following Breach of Duty: From Contract to Conscience’ [2010] LMCLQ 95.

93 Most famously *Boardman v Phipps* [1967] 2 AC 46 (HL); cf *Murad v Al-Saraj* [2005] EWCA Civ 959, [2005] WTLR 1573. This seems to have been the point of disagreement. Watts (n 82) seems of the view that the agent in *Imageview* was not dishonest enough to deserve punishment (or at least that there was no explicit finding of dishonesty by the agent), and that the undisclosed conflict of interest did not sufficiently affect the value of the performance contracted for and received by the principal.

94 Francis Reynolds and Peter Watts (eds), *Bowstead and Reynolds on Agency* (23rd edn, Sweet & Maxwell 2023).

95 Watts (n 82) 203.

96 ibid 205.

97 See eg *Rahme v Smith & Williamson Trust Corp Ltd* [2009] EWHC 911 (Ch); *Stupples v Stupples & Co (High Wycombe) Ltd* [2012] EWHC 1226 (Ch), [2013] 1 BCLC 729; *Avrahami v Biran* [2013] EWHC 1776 (Ch); *Hosking v Marathon Asset Management LLP* [2016] EWHC 2418 (Ch), [2017] Ch 157.

98 Reynolds and Watts (n 94) [7-050]: ‘Perhaps only the Supreme Court could now undertake a principled reconsideration of the law.’

99 [2009] EWHC 911 (Ch) [140]–[141].

100
*Avrahami v Biran* (n 97) [339]. Citing J McGhee (ed), *Snell’s Equity* (32nd edn, Sweet & Maxwell 2010) [7-062].

101 [2019] EWCA Civ 817, [2019] 3 All ER 429 [82], [91].

102 Citing approvingly *Andrews v Ramsa* (n 81) 638 (Lord Alverstone CJ).

103 See eg Burrows, *Remedies* (n 3); James Edelman, ‘Equitable Torts’ (2002) 10 TLJ 64.

104 cf Lionel Smith, ‘Deterrence, Prophylaxis and Punishment in Fiduciary Obligations’ (2013) 7 J Eq 18; Lionel Smith, *The Law of Loyalty* (OUP 2023).

105 Peter Birks, *Unjust Enrichment* (OUP 2004) 222: ‘the most important defence of all’; Andrew Burrows, *A Restatement of the English Law of Unjust Enrichment* (OUP 2012) 117 ‘in theory and in practice is the most important in the law of unjust enrichment … a general defence to unjust enrichment that can apply to almost all unjust factors’; Robert Stevens, ‘Defenses’ in Andrew Gold and others (eds), *Oxford Handbook of the New Private Law* (OUP 2020) 542: ‘By far the most significant defense’; Lionel Smith, ‘Defences and the Disunity of Unjust Enrichment’ in A Dyson, J Goudkamp and F Wilmot-Smith (eds), *Defences in Unjust Enrichment* (Hart Publishing 2016).

106
*Scottish Equitable Plc v Derby* [2001] EWCA Civ 369, [2001] 3 All ER 818 [30]; *Phillip Collins v Davis* [2000] 3 All ER 808, 827 (Parker J), applying *Lipkin Gorman* [1991] 2 AC 548 (HL) 580.

107
*Lipkin Gorman* (n 106). *Skandinaviska Enskilda Banken AB (Publ) v Conway* [2019] UKPC 36, [2019] 3 WLR 49 [94] said it is traceable as far back to Lord Mansfield’s judgment in *Moses v Macferlan* (1760) 2 Burrow 1005, 97 ER 676.

108
*Lipkin Gorman* (n 106) 580 (Lord Goff); see also 560 (Lord Templeman).

109 Andrew Burrows, ‘Summary and Foundations’ in ‘Review of Robert Stevens, *The Laws of Restitution*’ [2024] LMCLQ 353, 357.

110 To my present thinking, the most promising interpretation of an ‘enrichment-based’ rationale is that disenrichment is (i) necessary and (ii) *pro tanto* sufficient, although (iii) it is not all-things-considered sufficient. Thus, it can be ‘lost’ through the presence of ‘extra facts’, eg the defendant’s ‘disqualifying fault’. (It is not a mere ‘denial’.)

111 Elise Bant, *The Change of Position Defence* (Hart Publishing 2009) 163, 217. Bant advances a mixed rationale based on two distinct aims of ‘irreversibility protection’ and ‘prophylaxis’. See generally ch 6.

112 Birks (n 105) 208–9. For Birks, ‘security of receipts’ provided a second related rationale, on which see eg *Kleinwort Benson Ltd v Lincoln CC* [1999] 2 AC 349 (HL) 382; *Dextra Bank v Bank of Jamaica* [2002] 1 All ER (Comm) 193 (PC) [38].

113 [1991] 2 AC 548 (HL) 580. See generally P Mitchell, C Mitchell and S Watterson (eds), *Goff and Jones on The Law of Unjust Enrichment* (10th edn, Sweet & Maxwell 2022) ch 27, s 4.

114 On ‘wrongdoers’, a debate exists over change of position’s applicability to ‘restitution for wrongs’: see eg *Kuwait Airways v Iraqi Airways* [2002] UKHL, 19 [2002] 2 AC 883 [78]–[79] (Lord Nicholls); *Test Claimants in the FII Group Litigation* [2014] EWHC 4302, [2014] STC 1471 [309]–[315] (Henderson J). There is Singaporean authority suggesting that change of position is available even against a tortfeasor (ie a wrongdoer), but that he will be disentitled if he is a *culpable* wrongdoer, with ‘moral turpitude’: *Cavenagh Investments Pte Ltd v Kaushik Rajiv* [2013] SGHC 45, [2013] 2 SLR 543 [63]–[65]. See also Birks (n 105) 213.

115
*Niru Battery v Milestone Trading* [2003] EWCA Civ 1446, [2004] QB 985 [147]–[149], [162] (Clarke LJ), [182]–[185], [192] (Sedley LJ); *Haugesund Kommune v Depfa ACS Bank* [2010] EWCA Civ 579, [2012] QB 549 [152] (Etherton LJ); *Australian Financial Services & Leasing v Hills Industries* [2014] HCA 14, (2014) 253 CLR 560 [1] (French CJ), [65]–[77], [81] (Hayne, Crennan, Kiefel, Bell and Keane JJ), [155] (Gageler J).

116 cf *Harris v Digital Pulse* (n 23) [51]–[52] (Spigelman CJ): ‘Equity is concerned with the conscience of both parties, so that a balancing exercise is always required … a subsidiary principle to the effect that equity does not punish can be deduced, albeit not as a principle that will be applied in a rigid way.’ See also the explanation within n 23. Thanks to the anonymous reviewers for forcing me to confront this point.

117 Terminology coined by Stephen Perry, ‘The Moral Foundations of Tort Law’ (1991) 77 Iowa Law Review 449, 461.

118
*Dextra Bank* (n 112) [40]–[45]. cf *National Bank of NZ v Waitaki* [1999] 2 NZLR 211 (CA).

119
*Dextra Bank* (n 112) [45].

120
*Kelly v Solari* (1841) 9 M& W 54; 152 ER 24; *Scottish Equitable v Derby* (n 106) [17], [24]–[25], [34].

121 eg *ITC v HMRC* (n 55) [39]: ‘A claim based on unjust enrichment does not create a judicial licence to meet the perceived requirements of fairness on a case-by-case basis: legal rights arising from unjust enrichment should be determined by rules of law which are ascertainable and consistently applied’. See also *Lipkin Gorman* (n 106) 578; *BFC v Parc (Battersea) Ltd* [1999] 1 AC 221, 227; cf *Peter v Beblow* (1993) 1 SCR 980, 988.

122
*Abou-Rahmah v Abacha* [2006] EWCA Civ 1492, [2007] 1 All ER (Comm) 827 [88]; *Armstrong v Winnington* [2012] EWHC 10 (Ch), [2013] Ch 156 [110]; *Dextra Bank* (n 112) [45]; *Scottish Equitable v Derby* (n 106) [34]. cf *National Bank of NZ v Waitaki* (n 118), criticised in Birks (n 105) 216–19.

123
*Niru Battery Manufacturing Co v Milestone Trading* [2002] EWHC 1425 (Comm), [2002] 2 All ER (Comm) 705 [135] (Moore-Bick J) discussing *Twinsecta Ltd v Yardley* [2002] UKHL 12, [2002] 2 AC 164; cf *Barlow Clowes International Ltd v Eurotrust International Ltd* [2005] UKPC 37, [2005] 1 WLR 1453.

124 [2004] EWCA Civ 47, [2004] 1 WLR 2775. Other examples include *Niru Battery* EWHC (n 123); *Jones v Churcher* [2009] EWHC 722 (QB).

125
*Lipkin Gorman* (n 106) 580 (Lord Goff); *Niru Battery* EWHC (n 123) [135]: ‘Where he knows that the payment he has received was made by mistake, the position is quite straightforward: he must return it.’

126
*Cressman* (n 124) [1], [6]–[8].

127 ibid [21].

128 ibid [41].

129 Ripstein, *Private Wrongs* (n 2); John Goldberg and Benjamin Zipursky, *Recognising Wrongs* (2020); Peter Birks, ‘The Concept of a Civil Wrong’ in David Owens (ed), *The Philosophical Foundations of Tort Law* (OUP 1997).

130 See eg Gardner, ‘Wrongs and Faults’ (n 19) 54–61; John Gardner, ‘Obligations and Outcomes in the Law of Torts’ in Peter Cane and John Gardner (eds), *Relating To Responsibility* (OUP 2001); John Gardner, ‘Some Rule-of-Law Anxieties about Strict Liability in Private Law’ in Lisa Austin and Dennis Klimchuk (eds) *Private Law and The Rule of Law* (OUP 2014); AP Simester, *Fundamentals of Criminal Law: Responsibility, Culpability, and Wrongdoing* (OUP 2021) 17–19.

131 Hence the necessity of inserting ‘reasonable endeavours’ clauses: see eg *CPC Group Ltd v Qatari Diar Real Estate Investment Co* [2010] EWHC 1535 (Ch); *Sainsbury’s Supermarkets Ltd v Bristol Rovers (1883) Ltd* [2015] EWHC 2002 (Ch); *Gaia Ventures Ltd v Abbeygate Helical (Leisure Plaza) Ltd* [2019] EWCA Civ 823.

132 See eg *Reeves v Commissioner of Police* [2000] 1 AC 360 (HL) 371; *Corr v IBC Vehicles* [2008] UKHL 13, [2008] 1 AC 884, 905; Peter Cane, *The Anatomy of Tort Law* (Hart Publishing 1997) 58; Robert Stevens, ‘Should Contributory Fault be Analogue or Digital?’ in Andrew Dyson, James Goudkamp and Frederick Wilmot-Smith (eds), *Defences in Tort* (Hart 2015); Tettenborn and others (ed), *Clerk & Lindsell on Torts* (23rd edn, Sweet & Maxwell 2020) ch 3; cf James Goudkamp, *Tort Law Defences* (Hart Publishing 2016) 3, 7.

133 See ss 1, 4; *Reeves* (n 132) 382 (Lord Hope), 369 (Lord Hoffmann); *Standard Chartered Bank v Pakistan National Shipping Corp (No 2)* [2002] UKHL 43, [2003] 1 AC 959, 965 (Lord Hoffmann).

134
*Clerk & Lindsell* (23rd edn) (n 132). cf *Clerk & Lindsell on Torts* (24th edn, Sweet & Maxwell 2023) ch 26.

135 Torts (Interference with Goods) Act 1977, s 11(1).

136
*Co-operative Group (CWS) Ltd v Pritchard* [2011] EWCA Civ 329, [2012] QB 320 [33]–[34] (Aikens LJ), [78] (Smith LJ); *Lane v Holloway* [1968] 1 QB 379 (CA) 392 (Salmon LJ), 393 (Winn LJ); cf *Murphy v Culhane* [1997] QB 94 (CA).

137
*Standard Chartered Bank v Pakistan* (n 133); *Alliance And Leicester Building Society v Edgestop Ltd* [1993] 1 WLR 1462 (CA), 1477 (Mummery LJ); cf John Murphy, ‘Misleading Appearances in the Tort of Deceit’ (2016) CLJ 301.

138
*Corporacion Nacional del Cobre de Chile v Sogemin Metals Ltd* [1997] 1 WLR 1396 (Ch), 1405 (Carnwath J).

139 It does not explain *all* exclusions: see eg the Torts (Interference with Goods) Act 1977, which also excludes contributory negligence for ‘proceedings founded on conversion’, a famously strict tort: see eg *Fowler v Hollins* (1875) LR 7 QB 616, 639. This exclusion must be justified elsewise. Note, however, that contributory negligence is a defence available to bankers by s 47 of the Banking Act 1979, in addition to the statutory immunity under s 4 of the Cheques Act 1957. cf treatment of dishonest convertors: *Kuwait Airways* (n 114) [102], [104] (Lord Nicholls).

140 Compare text to n 117. Compare also John Gardner, ‘What Is Tort Law For? Part 2. The Place of Distributive Justice’ in John Oberdiek (ed), *Philosophical Foundations of the Law of Torts* (OUP 2014) 349.

141 Glanville Williams, *Joint Torts and Contributory Negligence* (Stevens & Sons 1951) 202. cf now *Letang v Cooper* [1965] 1 QB 232 (CA).

142 Williams (n 141). Compare James Goudkamp, ‘Contributory Negligence and Trespass to the Person’ (2011) 127 LQR 518, 520–1.

143
*Co-operative Group v Pritchard* (n 136) [33]–[35], [37].

144 Goudkamp (n 142) 521.

145
*Pasley v Freeman* (1789) 3 TR 51, 100 ER 450; *Derry v Peek (*1889) 14 App Cas 337 (HL).

146
*Standard Chartered Bank v Pakistan* (n 133) [16]. See also Nick McBride, *Humanity of Private Law II* (Hart Publishing 2020) 108–16.

147 Fraudsters do not benefit from the normal requirement to show ‘but for’. *Standard Chartered Bank v Pakistan* (n 133) [19]. See also [15]: ‘The law simply ignores the other reasons why he paid’, discussing *Edgington v Fitzmaurice* (1885) 29 Ch D 459. See also *Zurich Insurance Co Plc v Hayward* [2016] UKSC 48, [2017] AC 142; *BV Nederlandse Industrie van Eiprodukten v Rembrandt Enterprises Inc* [2019] EWCA Civ 596, [2019] 3 WLR 1113.

148 Solène Rowan, ‘Punishment and Private Law: Some Comparative Observations’ in E Bant and others (n 1) 66. Its predecessor before the 2016 reforms was art 1150 of the 1804 Civil Code.

149 Sandy Steel, ‘Culpability and Compensation’ in James Goudkamp, Mark Lunney and Leighton McDonald (eds), *Taking Law Seriously: Essays in Honour of Peter Cane* (Hart Publishing 2021) 65; Burrows, *Remedies* (n 3) ch 7; Burrows, ‘Summary and Foundations’ (n 109) 355.

150
*The Wagon Mound* [1961] AC 388 (PC); *cf In Re Polemis* [1921] 3 KB 560 (CA).

151
*Doyle v Olby* [1969] 2 QB 158 (CA); affirmed and applied in *Smith New Court Securites Ltd v Citibank NA* [1997] AC 254 (HL). Hence the controversy over its application to Misrepresentation Act 1967, s 2(1) and the ‘fiction of fraud’: *Royscot v Rogerson* [1991] 2 QB 297 (CA).

152
*Doyle v Olby* (n 151) 167.

153 ibid 282–3 (emphasis added).

154 There is no prospect of ‘sharing’ or ‘apportionment’ between a fraudster and his victim; instead, the disentitlement is complete and appears like a rule-like blanket exclusion, so the distributive explanation is, as above, implausible for these cases: text to n 140. On the dangers of ‘risk-distributive’ justice see also Gardner, ‘What Is Tort Law For?’ (n 140) 350–3.

155 On culpability’s role in tort more generally see Steel (n 149).

156 Goudkamp and Katsampouka, ‘Place of Punishment’ (n 1) 1275.

157 ibid 1271–7; See also *Reeves* (n 132) 394.

158
*Quinn v Leathem* [1091] AC 495 (HL), 537 (Lord Lindley).

159
*Kuwait Airways* (n 114) 102]–[104] (Lord Nicholls).

160
*Essa* v *Laing Ltd* [2004] EWCA Civ 2, [2004] ICR 746.

161
*Jones* v *Ruth* [2011] EWCA Civ 804, [2012] 1 WLR 1495.

162 (1880) 5 App Cas 25 (HL) 39.

163 ibid (emphasis added).

164
*Rahme v Smith* (n 97) [140] (Morgan J).

165 See eg *Rookes v Barnard* (n 27) (Lord Devlin) 1221: ‘this confuses the civil and criminal functions of the law … an anomaly [in] the law of England’.

166 Rawls (n 20).

167 Weinrib (n 2); Ripstein, *Private Wrongs* (n 2); Beever (n 2); Stevens, *Torts & Rights* (n 2).

168 See eg *Rookes v Barnard* (n 27) 1227 (Lord Devlin); *Cassell v Broome* (n 27) 1087 (Lord Reid), 1100 (Lord Morris), 1114 (Lord Wilberforce), 1128 (Lord Diplock), 1135 (Lord Kilbrandon); *Whiten* (n 27) [158] (Lebel J).

169 See eg Feinberg (n 20) 423.

170 Hence the move made by Stevens, *Torts & Rights* (n 2) 85. For other versions of ‘retributivism’, see also Pey-Woan Lee, ‘Contract Damages, Corrective Justice and Punishment’ (2007) 70 MLR 887; Peter Cane, ‘Retribution, Proportionality, and Moral Luck in Tort Law’ in Cane and Stapleton (eds), *The Law of Obligations* (OUP 1998) esp 169–72. cf E Encarnacion, ‘Resilience, Retribution, and Punitive Damages’ (2021) 100 Tex L Rev 1025, whose version of ‘retribution’ *requires* a plaintiff to be left better off, based on his novel idea of a ‘resilience interest’ (and with whom I thus have to disagree).

171
*Whiten* (n 27) [156]

172
*Cassell v Broome* (n 27) 1086–7.

173 ibid.

174 Penner, ‘Punishments and Penalties’ (n 2) 117–18.

175 See eg *Whiten* (n 27) [151]; Arthur Ripstein, *Equality, Responsibility and the Law* (CUP 1999) 140; John Gardner, *Offences and Defences* (OUP 2007) 221–34. In tort, see eg Cane, ‘Retribution’ (n 170) 143–4, 160–1, 169–70: ‘one of the functions of damages awards in tort is to express disapproval of and to discourage certain kinds of tortious conduct. Viewed in this way, even compensatory damages may have a punitive aspect … The only question is whether we think that punitive damages express a degree of disapproval and discouragement out of proportion to the seriousness and culpability of any conduct which we call tortious. Ultimately, it seems to me that a judgment that they are disproportionate to tortious conduct is the only basis on which we could pronounce punitive damages, or disgorgement damages based on deliberate gain-seeking, not properly part of tort law.’

176 Goudkamp and Katsampouka, ‘Place of Punishment’ (n 1) 1276, 1291.

177 ibid.

178 In the case of a defence, it is capped at the maximum value of the claim against the defendant, assuming the defence reduces none of its value.

179 cf Forfeiture Act 1982, s 2. Discussed above, text to n 65 and onwards.

180 cf n 176; Steel (n 149) 68–9.

181 eg *Smith v Leech Brain* [1962] 2 QB 405. Cane, ‘Retribution’ (n 170); Waldron, ‘Moments of Carelessness and Massive Loss’ in Owen (n 129); James Penner, ‘Don’t Crash into Mick Jagger When He Is Driving His Rolls Royce: Liability in Damages for Economic Loss Consequent upon a Personal Injury’ in Miller and Oberdiek (eds), *Civil Wrongs and Justice in Private Law* (OUP 2020) 253–4.

182 David Lewis, ‘The Punishment that Leaves Something to Chance’ (1989) 18 Philosophy and Public Affairs 53. See also Waldron (n 181) 401–5; Steel (n 149) 68–9; Andrew Ashworth, ‘Taking the Consequences’ in Shute, Gardner and Horder (eds), *Action and Value in Criminal Law* (OUP 1993); James Edwards and AP Simester, ‘Crime, Blameworthiness, and Outcomes’ (2019) 39 OJLS 50.

183 Though not ‘future losses’. Pinning down a lump sum at trial may require estimations about post-trial events. But ‘provisional damages’ can be awarded for lost earnings due to personal injury: Damages Act 1996, s 2A; Senior Courts Act 1981, s 32A.

184 Some defences are arguably not conduct-guiding: compare Meir Dan-Cohen, ‘Decision Rules and Conduct Rules’ (1984) 97 Harv L Rev 625; Gardner, *Offences and Defences* (n 175) 138; Stephen Smith, *Rights, Wrongs, Injustices* (OUP 2019) ch 9.

185 Frederick Wilmot-Smith and Lord (Phillip) Sales, ‘Justice for Foxes’ (2022) 138 LQR 583, 588–94.

186 NB the diminishing marginal utility of wealth.

187 See n 4.

188 Feinberg (n 20) 418. cf Penner, ‘Punishments and Penalties’ (n 2); on ‘civil penalties’ and proportionality constraints, see Bant and Paterson (n 23).

